# Long-Range Mid-Infrared Energy Transfer Mediated by Hyperbolic Phonon Polaritons

**DOI:** 10.1021/acs.nanolett.6c01042

**Published:** 2026-05-28

**Authors:** Gonzalo Álvarez-Pérez, Simone De Liberato, Huatian Hu

**Affiliations:** † Center for Biomolecular Nanotechnologies, Istituto Italiano di Tecnologia, Via Barsanti 14, 73010 Arnesano, Italy; ‡ 96976 Istituto di Fotonica e Nanotecnologie, Consiglio Nazionale delle Ricerche (CNR), Piazza Leonardo da Vinci 32, Milano 20133, Italy; § School of Physics and Astronomy, University of Southampton, Southampton SO17 1BJ, United Kingdom

**Keywords:** quantum electrodynamics, dipole−dipole interactions, two-dimensional materials, canalization, twistoptics, quantum emitters

## Abstract

We provide a framework to theoretically describe long-range energy transfer in single and twisted two-dimensional hyperbolic slabs. We demonstrate that phonon polaritons, quantum superpositions of photons and lattice vibrations in polar dielectrics, can mediate and enhance energy transfer at ranges far exceeding those of conventional mid-infrared (MIR) platforms and with extreme directionality. This is because the dipole–dipole interaction potential energy diverges along the asymptotes of the real-space hyperbolic opening angle. Our findings allow us to extend classical and quantum interactions between dipoles, typically strictly confined to the near-field, beyond several free-space MIR wavelengths. We use α-MoO_3_ as a representative material, but this mechanism could be extended to other anisotropic media beyond the MIR.

## Graphical abstract

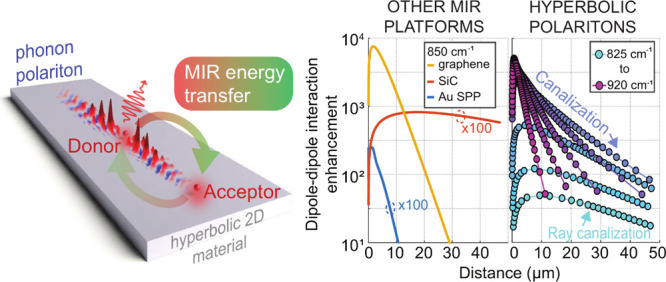

Energy transfer between localized excitations, such as atoms, molecules, or spins, is a universal process governing interactions across the electromagnetic spectrum. It can occur radiatively through photon emission and reabsorption or nonradiatively via direct coupling. Resonant energy transfer stands out as a fundamental pathway in which a donor transfers energy nonradiatively to an acceptor through so-called dipole–dipole interactions (DDIs), mediated by virtual photon exchange. DDIs underpin a broad range of phenomena: from Casimir and van der Waals (vdW) forces,^1,2^ superradiance and entanglement in atomic ensembles,^3–5^ and Förster energy transfer (FRET) between molecules or quantum dots,^6–8^ to cooperative shifts in superconducting qubits and magnonic systems.^9–13^

However, their strength decays rapidly with distance, scaling as 1/*r* ^3^ in the near field and 1/*r* in the far field,^14^ restricting coherent coupling to subwavelength separations. Extending their range requires a mediator capable of carrying electromagnetic energy beyond the donor’s near field. Two main strategies are typically employed for this: tuning the emitters’ intrinsic properties (e.g., Rydberg atoms, superconducting qubits),^15–17^ or engineering the mediating platforms to tailor propagation and coupling, using cavities, waveguides, or metamaterials.^14,18–22^ These mediating platforms, summarized in Figure 1, span all spectral regimes and show the fundamental trade-offs between field enhancement, interaction length, and directionality. Vacuum near-fields dominate at subwavelength scales, providing strong but highly localized coupling. Cavities and photonic crystals enhance coherence through mode confinement but remain spectrally narrow, while waveguides enable long-range and directional energy transfer with moderate field enhancement. Importantly, collective excitations can also act as frequency-specific mediators: magnons in the radiofrequency (RF) to the microwave range, acoustic phonons in the terahertz (THz) domain, graphene plasmons and surface plasmon polaritons (SPPs) in the MIR to the visible, and exciton polaritons in the visible to the UV. In particular, localized surface plasmons (LSPs) can strongly enhance near fields at visible frequencies but are limited to interaction lengths of *d*/λ_0_ ∼ 0.1–1.^23–25^ Plasmonic nanowires can guide energy transfer at visible frequencies over interaction lengths of *d*/λ_0_ ∼ 5, which however remains limited to a few micrometers in absolute terms.^26,27^ SPPs and graphene plasmons can extend the interaction range but with a lower confinement with respect to LSPs and less directional control than waveguides.^8,28–32^ As such, no existing platform simultaneously achieves strong confinement, long-range interaction, and high directionality.

**Figure 1:**
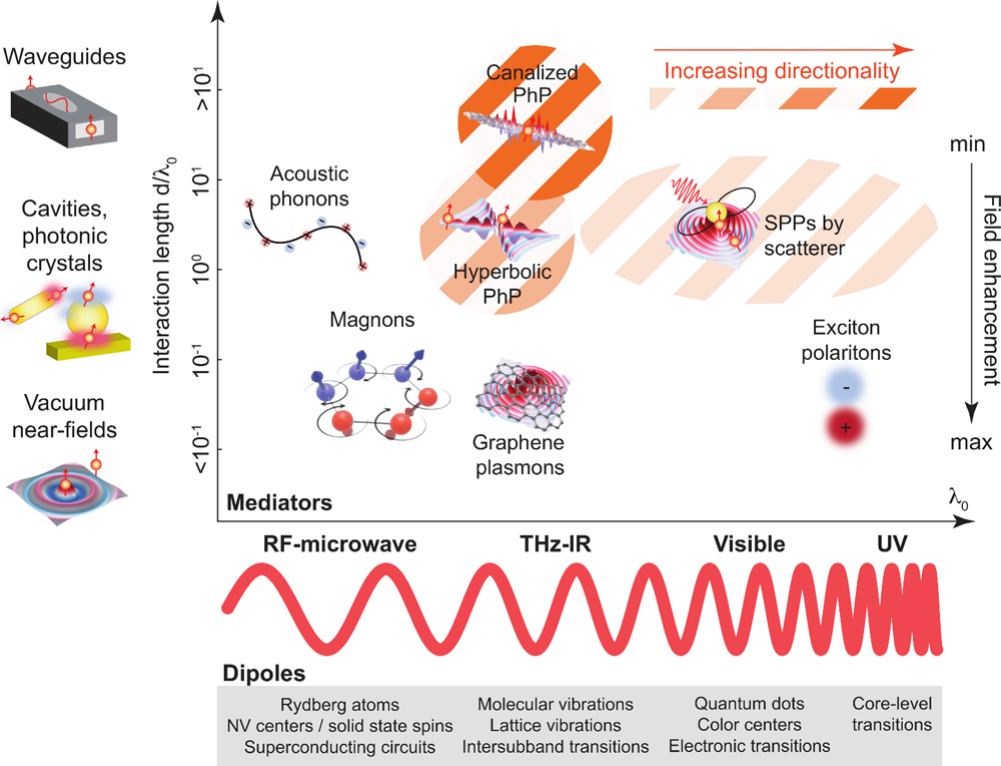
Landscape of platforms for long-range DDIs across the electromagnetic spectrum. DDI mediators are compared by their normalized interaction length *d*/λ_0_. Platforms are arranged by operating frequency. The orange stripes indicate directionality.

These limitations are particularly acute in the MIR (3–20 μm), where light couples to molecular vibrations and infrared-active phonons rather than to electronic or magnonic excitations. In this regime, energy exchange occurs in the form of vibrational energy transfer (VET), which is governed by vibrational DDIs.^14,33,34^ Energy can be coherently exchanged between vibrational excitations despite they are typically thermally populated and short-lived at room temperature. While efficient quantum emitters are scarce in this range, coupling molecular vibrations, intersubband transitions, or optical phonons via long-range DDIs offers a promising route to control the infrared energy flow. Yet, like their visible and RF-microwave counterparts, MIR DDIs decay rapidly with distance, preventing coherent coupling between spatially separated dipoles. Existing mediating strategies fail to overcome the aforementioned limitations: metal-based plasmons suffer from high losses and poor confinement, graphene plasmons have stronger confinement but still have rather high ohmic losses, dielectric resonators offer modest enhancement without directionality, boron nitride nanowires improve directionality but still with limited enhancement, and microcavities are constrained by narrow bandwidths and fabrication complexity.^8,28^ Consequently, enhancing MIR DDIs beyond the near field remains an open challenge.

In this context, phonon polaritons (PhPs) in polar dielectrics offer a promising solution. These hybrid excitations, arising from the strong coupling between infrared photons and optical phonons, combine deep subwavelength confinement, low optical losses, and strong field enhancement in the MIR.^35^ Over the past decade, PhPs in low-symmetry polar dielectrics have attracted considerable interest within the MIR nanophotonics community owing to their ability to support extreme confinement and highly directional (or, more precisely, hyperbolic) propagation.^36–38^ Importantly, PhPs spectrally overlap with the vibrational modes of numerous molecular species, enabling vibrational strong coupling. This phenomenon has been experimentally demonstrated in several studies, both in the far field^39^ and the near field,^40,41^ allowing remote^42^ and on-chip^43^ detection of molecular vibrations. Moreover, hyperbolic PhPs in graphene/hBN heterostructures can transfer a significant fraction of the injected electronic and thermal power out of plane via MIR radiation.^44^ This highlights the potential of hyperbolic PhPs for strong light-matter interactions with low losses, but their capabilities as long-range energy mediators remain unexplored.

Here, we theoretically demonstrate that hyperbolic PhPs can in fact serve as exceptional mediators of long-range DDIs in the MIR regime, as they combine strong field confinement, low optical losses, pronounced directionality and large local density of optical states (LDOS). These properties enable vibrational dipoles to couple over tens of micrometres, distances exceeding 5λ_0_ and orders of magnitude larger than the PhP wavelength in the MIR. Remarkably, the DDI strength is enhanced by factors greater than 10^3^ compared with gold- or SiC-based platforms, and even surpasses that achieved with graphene plasmons. Moreover, we show that twisting α-MoO_3_ layers allows to tune the dipole–PhP coupling and thus long-range DDIs in the MIR. Our formalism could be extended to other strongly anisotropic materials beyond the MIR.^45,46^

To understand the physical origin of such long-range DDIs, we note that the strong structural anisotropy inherent to some vdW materials gives rise to highly direction-dependent optical responses.^47^ In these media, light propagation is described by a frequency-dependent dielectric tensor *ε̃*(ν). Hereafter, ν denotes the frequency in wavenumbers and tildes denote tensors. Here, we consider biaxial materials, which include many prototypical strongly anisotropic MIR media and are characterized by three distinct principal permittivities along orthogonal crystal axes *ε̃*(ν) = diag­[*ε*_*x*_(ν), *ε*_*y*_(ν), *ε*_*z*_(ν)].^48–50^ Importantly, the signs of the in-plane permittivity components *ε*_*x*,*y*_(ν) mainly determine the propagation along the material’s surface. This can be visualized by plotting the isofrequency curve (IFC), i.e., a slice of the polariton dispersion ν­(**k**) with in-plane wavevector **k** = (*k*_*x*_, *k*_*y*_) at a constant frequency. When *ε*_*x*_ = *ε*_*y*_, propagation is isotropic and the IFC is circular (Figure 2b, left), with the Poynting vector **S**, perpendicular to the IFC,^51,52^ indicating uniform in-plane propagation. When *ε*_*x*_ and *ε*_*y*_ have opposite signs (within the reststrahlen bands bounded by TO and LO phonons^53^), the IFC becomes hyperbolic, restricting propagation to specific directions (Figure 2b, middle). Such in-plane hyperbolic behavior occurs naturally in several vdW^45,46,54,55^ and bulk^56,57^ crystals, as well as in hyperbolic metamaterials.^2,58,59^ Wavevectors near the hyperbola’s asymptotes correspond to propagation along a single direction, yielding a high LDOS. Interestingly, stacking in-plane hyperbolic vdW layers at different twist angles allows to control the polariton hybridization and propagation, from the MIR^60–63^ down to the terahertz.^64^ At a specific critical twist angle, the so-called *magic* angle, the IFC flattens, leading to canalization, defined by all the allowed modes propagating collinearly without diffraction (Figure 2b, right). This regime has been demonstrated in twisted bilayers^60–63^ and trilayers^65^ of α-MoO_3_, a paradigmatic MIR in-plane hyperbolic material.

**Figure 2:**
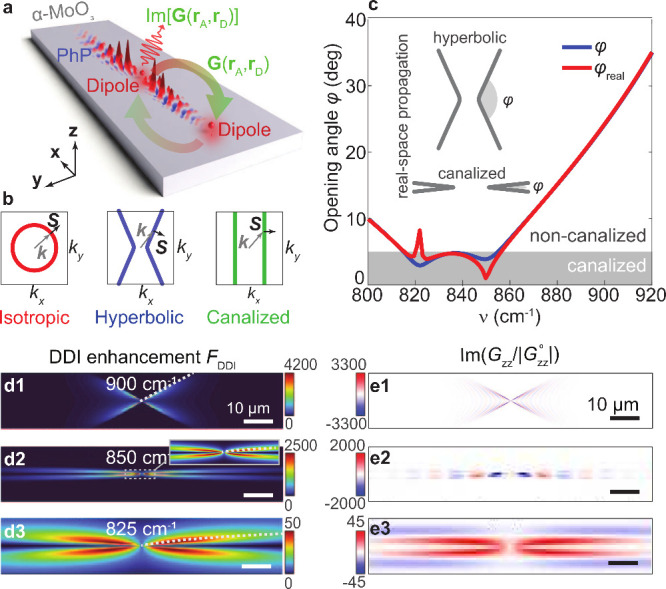
Long-range MIR DDIs mediated by hyperbolic PhPs in a single α-MoO_3_ slab. (a) Two dipoles on a 200 nm-thick α-MoO_3_ slab, interacting via PhPs described by the DGF **G̃**(**r**_*A*_, **r**_*D*_). (b) IFCs for isotropic (left), hyperbolic (middle), and canalized (right) PhPs; gray and black arrows indicate **k**_*p*_ and the propagation direction **S**. (c) Opening angle φ of hyperbolic PhP propagation from 800 to 920 cm^–1^, calculated using complex (blue) and real-part (red) permittivities; canalization occurs around 820–850 cm^–1^. (d1–d3) Normalized DDI enhancement *F*_DDI_ at 900, 850, and 825 cm^–1^ for a 200 nm α-MoO_3_ slab. (e1–e3) Normalized imaginary part Im­[*G*_*zz*_/|*G*_*zz*_^0^|] at the same frequencies, showing phase and LDOS patterns.

Figure 2a illustrates two dipoles, a donor and an acceptor supporting MIR vibrational modes and coupled via PhPs on a α-MoO_3_ slab. The dipoles are located at **r**_*j*_ (*j* = *D*, *A*) and have transition frequencies ω_*j*_ and transition dipole moments **μ̂**_*j*_, with hats denoting operators hereafter. The donor’s excitation can be nonradiatively transferred to the acceptor via DDIs,^66^ without photon emission. The resonant DDI potential energy governing the energy transfer between the donor and the acceptor can be derived within quantum electrodynamics (QED) theory. Specifically, the interaction between two dipoles located on a slab of a biaxial material can be described through the Hamiltonian

$${Ĥ}_{\mathrm{int}}=-\underset{j}{\sum }\int_{0}^{\infty }d\omega \left[{\mathrm{\mu ̂}}_{j}\cdot Ê\left(r_{j},\omega \right)+\mathrm{H.c.}\right]$$

where H.c. denotes the Hermitian conjugate and ω is the angular frequency. The quantized electric field in an absorbing, dispersive medium is then expressed in terms of bosonic operators *â*(**r**, ω) and *â* ^†^(**r**, ω), which create and annihilate polaritonic excitations:

$$Ê\left(r_{A},\omega \right)=i\sqrt{\frac{\hbar }{\pi \varepsilon_{0}}}\frac{\omega^{2}}{c^{2}}\int d^{3}r_{D}\sqrt{Im\mathrm{\varepsilon ̃}}\mathrm{G̃}\left(r_{A},r_{D};\omega \right)\cdot â\left(r_{D},\omega \right)$$

where *ε*_0_ is the vacuum permittivity, *c* is the speed of light, and **G̃**(**r**_*A*_, **r**_*D*_; ω) is the dyadic Green’s function (DGF), a symmetric 3 × 3 tensor that governs field propagation.^66^

Each dipole (*D* or *A*) is treated as a two-level system with ground and excited states |*g*_*A,D*_⟩ and |*e*_*A,D*_⟩. We define the lowering and raising operators σ̂_*j*_ = |*g*_*j*_⟩⟨*e*_*j*_| and σ̂_*j*_^†^ = |*e*_*j*_⟩⟨*g*_*j*_|. The electric dipole operator is **μ**_*j*_σ̂_*j*_ + **μ**_j_^*^σ̂_*j*_^†^, where **μ**_*j*_=⟨*g*_*j*_|μ̂_*j*_|*e*_*j*_⟩ is the transition dipole moment vector. For a donor at **r**_*D*_, the interaction Hamiltonian is ${Ĥ}_{D}=-{\mathrm{\mu ̂}}_{D}\cdot Ê\left(r_{D}\right)$ , and its de-excitation is described by the quantum current density operator **ĵ**(**r**_*A*_; ω) = –*iωδ*(**r**_*A*_, **r**_*D*_)**μ**_*D*_σ̂_*D*_, where **μ**_*D*_ = ⟨*g*_*D*_|**μ̂**_*D*_|*e*_*D*_⟩ is the donor’s transition dipole moment. It is convenient to determine the dipole moment of a real donor material from its absorption cross-section.^66^ For the sake of generality and to assess the performance of the PhP platform, we normalized the dipole moment, therefore considering perfect donors and acceptors. Within macroscopic QED, the resulting field at the acceptor’s position is $Ê\left(r_{A}\right)=\frac{\omega^{2}}{\varepsilon_{0}c^{2}}\mathrm{G̃}\left(r_{A},r_{D};\omega \right)\cdot \mu_{D}{\mathrm{\sigma ̂}}_{D}$ . For the donor initially excited (⟨σ̂_*D*_⟩ = 1), and taking the expectation value, this reduces to the classical dipole field: $E\left(r_{A}\right)=\frac{\omega^{2}}{\varepsilon_{0}c^{2}}\mathrm{G̃}\left(r_{A},r_{D};\omega \right)\cdot \mu_{D}$ . The effective DDI potential energy *V*_dd_ arises from the exchange of a virtual photon during the joint transition |*e*_*D*_, *g*_*A*_⟩ → |*g*_*D*_, *e*_*A*_⟩, where the donor de-excites and the acceptor is excited. Substituting ${Ĥ}_{A}=-{\mathrm{\mu ̂}}_{A}\cdot Ê\left(r_{A}\right)$ and evaluating the frequency integral yields^66^

1 $$V_{\mathrm{dd}}\left(r_{A},r_{D};\omega \right)=\frac{\omega^{2}}{\varepsilon_{0}c^{2}}\mu_{A}\cdot \mathrm{G̃}\left(r_{A},r_{D};\omega \right)\cdot \mu_{D}$$

Here, **μ**_*D*_ and **μ**_*A*_ are the donor and acceptor dipole moments, which we take as real. The real and imaginary parts of the DGF govern coherent and dissipative dynamics, respectively.^67^ Off-resonant terms enable nonlinearities and entangling gates, while near-resonant decay leads to super- and subradiant states that can guide or protect quantum information.^67^ The emitter’s decay rate is determined by Im­{**G̃**}.^32^ In hyperbolic media, the DDI potential can also be written as $V_{\mathrm{dd}}=\hbar \left(J_{\mathrm{dd}}-i\frac{\gamma_{\mathrm{dd}}}{2}\right)$ , where *J*_dd_ represents coherent energy exchange exchange through virtual photons and γ_dd_ accounts for super- or subradiant effects.^2^

As a representative MIR hyperbolic material, we consider a 200 nm-thick α-MoO_3_ slab, which supports highly confined TM-polarized PhPs^68^ (Figure 2a). Since s-SNOM measurements^69^ mainly probe *p*-polarized components, we analyze two *z*-polarized dipoles. *G*_*zz*_ and *G*_*zz*_^0^ are the *zz*-components of the DGFs in the α-MoO_3_ slab and in vacuum, respectively. Following ref 70, we model the slab as a two-dimensional (2D) conductivity layer (valid when *d* ≪ λ_PhP_ ^68^), which avoids calculating fields inside the slab. The two-point DGF reads:

2 $$G_{zz}\left(r_{A},r_{D}\right)=\frac{\frac{3}{\varepsilon^{2}}\left(\varepsilon_{x}^{2}\Delta y^{2}+\varepsilon_{y}^{2}\Delta x^{2}\right)e^{-q_{p}k_{0}H}}{\sqrt{\pi d}\varepsilon_{x}^{2}\varepsilon_{y}^{5/4}{\left(k_{0}d\right)}^{2}{\left(\varepsilon_{x}\Delta y^{2}+\varepsilon_{y}\Delta x^{2}\right)}^{5/4}}e^{i\left(\frac{2\varepsilon \sqrt{\varepsilon_{x}\Delta y^{2}+\varepsilon_{y}\Delta x^{2}}}{\varepsilon_{x}\sqrt{\varepsilon_{y}}d}-\frac{\pi }{4}\right)}$$

where ε is the permittivity of the medium surrounding the 2D layer and *k*_0_ = ω/*c* and *q*_*p*_ = *k*_*p*_/*k*_0_ is the normalized in-plane momentum:^70^

3 $$q_{p}=\frac{-2\varepsilon \sqrt{\varepsilon_{x}^{2}\Delta y^{2}+\varepsilon_{y}^{2}\Delta x^{2}}}{\varepsilon_{x}\sqrt{\varepsilon_{y}k_{0}d}\sqrt{\varepsilon_{x}\Delta y^{2}+\varepsilon_{y}\Delta x^{2}}}$$

The DDI potential energy enhancement, *F*_DDI_, can then be calculated as

4 $$F_{\mathrm{DDI}}=\frac{\left|V_{\mathrm{dd}}\left(r_{A},r_{D};\omega \right)\right|}{\left|V_{\mathrm{dd}}^{0}\left(r_{A},r_{D};\omega \right)\right|}=\frac{\left|G_{zz}\left(r_{A},r_{D};\omega \right)\right|}{\left|G_{zz}^{0}\left(r_{A},r_{D};\omega \right)\right|}$$

and the energy transfer rate scales as Γ_ET_/Γ_ET_^0^ ∝ *F*_DDI_^2^.^32^

We set the donor and acceptor height over the slab to *H* = 20 nm, and assume the half-spaces over and under the slab to be filled by vacuum (*ε* = 1). We focus on the spectral range 800–920 cm^–1^ (12.5–10.87 μm), where *ε*_*x*_ < 0 and *ε*_*y*_ > 0, leading to in-plane hyperbolic dispersion^50^ (see Supplementary Section I). The propagation angle $\varphi =\arctan\left(i\sqrt{\varepsilon_{y}/\varepsilon_{x}}\right)$ (or φreal=arctan[iRe(εy)/Re(εx)] )^68,71,72^ describes the opening of the hyperbolic rays (Figure 2b). Between 800 and 920 cm^–1^, canalization occurs at lower frequencies, while φ increases roughly linearly above 860 cm^–1^. Two minima in φ appear at 825 and 850 cm^–1^.

Figure 2d1–d3 displays *F*_DDI_, the normalized DDI enhancement, for various frequencies, obtained from eq 4 (a comparison with full-wave numerical simulations is shown in Supplementary Section II, in excellent agreement). The first dipole is fixed at the center, and the second varies along the *x*–*y* plane. *F*_DDI_ ≤ 1 indicates weaker coupling than in vacuum. At ν_0_ = 900 cm^–1^ (Figure 2d1), *F*_DDI_ peaks along the hyperbolic asymptotes (*F*_DDI_ ≈ 4200). To illustrate the mechanism for this enhancement, let us rewrite the last term in the denominator of eq 2 as *ε*_*x*_ *Δy* ^2^ + *ε*_*y*_ *Δx* ^2^ = *r* ^2^ cos^2^φ­(*ε* ^*x*^ tan^2^φ + *ε*_*y*_). As the in-plane angle φ approaches the opening angle $\varphi \to \varphi_{c}=\arctan\left(i\sqrt{\varepsilon_{y}/\varepsilon_{x}}\right)$ , we have *r* ^2^ cos^2^φ­(*ε*_*x*_ tan^2^φ + *ε*_*y*_) → *r* ^2^ cos^2^φ [*ε*_*x*_(−*ε*_*y*_/*ε*_*x*_) + *ε*_*y*_] = 0. As such, as φ → φ_*c*_, *G*_*zz*_(**r**_*A*_ – **r**_*D*_) → ∞ and therefore *V*_dd_(**r**_*A*_ – **r**_*D*_) → ∞. Consequently, the in-plane hyperbolicity of the system leads to a divergence of the DDI potential energy along the real-space hyperbolic opening angle φ_*c*_, even if losses are higher along this direction (see Supplementary Section III). The imaginary part Im­(*G*_*zz*_/*G*_*zz*_^0^) (Figure 2e1) reflects the PhP phase and LDOS enhancement, peaking along the asymptotes. At ν_0_ = 850 cm^–1^ (Figure 2d2), *F*_DDI_ reaches ∼2500, lower but with longer interaction distances due to reduced Im­(*ε̃*). Here, Re­(*ε*_*x*_) ≈ −22 and Re­(*ε*_*y*_) ≈ 0, corresponding to an epsilon-near-zero (ENZ) point that induces canalized propagation along *x*.^73^ Although the enhancement is weaker, the strong directionality enables controlled nanoscale energy transport (Figure 2e2). At ν_0_ = 825 cm^–1^ (Figure 2d3), *F*_DDI_ ≈ 50. The permittivities are *ε*_*x*_ = 127 + 109*i* and *ε*_*y*_ = – 0.6 + 0.12*i*, producing highly directional canalization along *x*. Canalization at this frequency cannot be explained by the real permittivity components alone and is instead a consequence of material losses. As such, this regime has been coined in the literature as *loss-induced canalization*.^74,75^ Moreover, the phase remains nearly constant along the canalization direction, leading to what has been named *ray canalization* (Figure 2e3). At this frequency, the longest-range coupling is achieved with minimal decay, though at the cost of reduced enhancement.

This trade-off between strength and range is summarized in Figure 3, which further shows that hyperbolic PhPs in α-MoO_3_ (825–920 cm^–1^) provide a promising platform to mediate strong and ultralong-range DDIs in the MIR, far surpassing conventional MIR platforms such as bare gold and SiC 200 nm-thick slabs, or a single graphene monolayer (*E*_*F*_ = 0.3 eV). Hyperbolic PhP-mediated DDIs are strong and can extend up to several PhP wavelengths (≈ λ_0_ = 10 μm). At lower frequencies, the enhancement weakens but persists over ∼50 μm (≈ 5λ_0_), where *F*_DDI_ > 10. The optimal balance occurs at 850 cm^–1^, providing a 1000-fold enhancement and 5λ_0_ range. Analytical calculations of the interaction length confirm this trend and show that the dependence on the dipole height *H* is weak (see Supplementary Section IV). For even longer-range interactions (>50 μm), ray canalization (825–840 cm^–1^) becomes advantageous despite its lower short-range enhancement.

**Figure 3:**
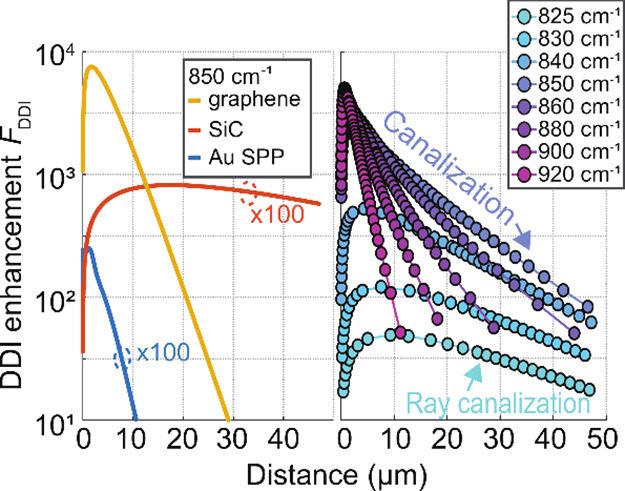
Benchmarking long-range MIR DDIs mediated by hyperbolic PhPs in a single α-MoO_3_ slab against existing MIR platforms. (Left) *F*_DDI_ for SPPs on Au, SPhPs in SiC, and graphene plasmons. (Right) *F*_DDI_ in a 200 nm α-MoO_3_ slab along the direction of maximum enhancement in the 825–920 cm^–1^ range.

Interestingly, eq 4 can be expressed as *F*_DDI_^0^ exp­(−*R*/*L*_*p*_) (see Supplementary Section V), where *R* is the distance, *L*_*p*_ the interaction length, and *F*_DDI_^0^ denotes the local enhancement of the DDI potential energy at the dipole position, with its real part corresponding to the Lamb shift and its imaginary part to the Purcell factor.^66^ The quantity *F*_DDI_^0^ exp­(−*R*/*L*_*p*_) thus completely encapsulates the aforementioned trade-off between long-range coupling and DDI potential enhancement. By integrating the *F*_DDI_ curves shown in Figure 3, we obtain single data points that capture both, revealing that the optimal configuration occurs at 850 cm^–1^ (see Supplementary Figure 5).

A direct experimental signature of PhP-mediated DDI enhancement is the reduction of the donor’s excited-state lifetime as the acceptor is positioned along the hyperbolic asymptotes. By properly placing the acceptor and measuring the donor’s photoluminescence lifetime (for instance, by time-resolved spectroscopy), one can map *F*_DDI_ directly. The large, predicted interaction lengths (>50 μm at 825 cm^–1^) make such measurements accessible with standard infrared microscopy setups. Order-of-magnitude estimates for net energy transfer rates and efficiencies using representative MIR emitters, including finite linewidths, detuning, dephasing, nonradiative decay, and spectral overlap are provided in Supplementary Section VI.

Given the exceptionally strong long-range DDIs mediated by hyperbolic PhPs in single slabs, we now study DDIs in two slabs twisted at an angle θ (Figure 4a), using α-MoO_3_ as a representative example. To accurately compute the DDI enhancement factor *F*_DDI_, we perform full-wave 3D finite-element method (FEM) simulations (Comsol Multiphysics), since 2D approximations fail to capture the out-of-plane coupling relevant in twisted systems.^61,65^ Each slab is 100 nm thick, matching the 200 nm single-layer case in Figure 2. We evaluate *F*_DDI_ at ν_0_ = 910 cm^–1^ (≈11 μm) for three twist angles: θ = 0°, 69.3°, and 90°.

**Figure 4:**
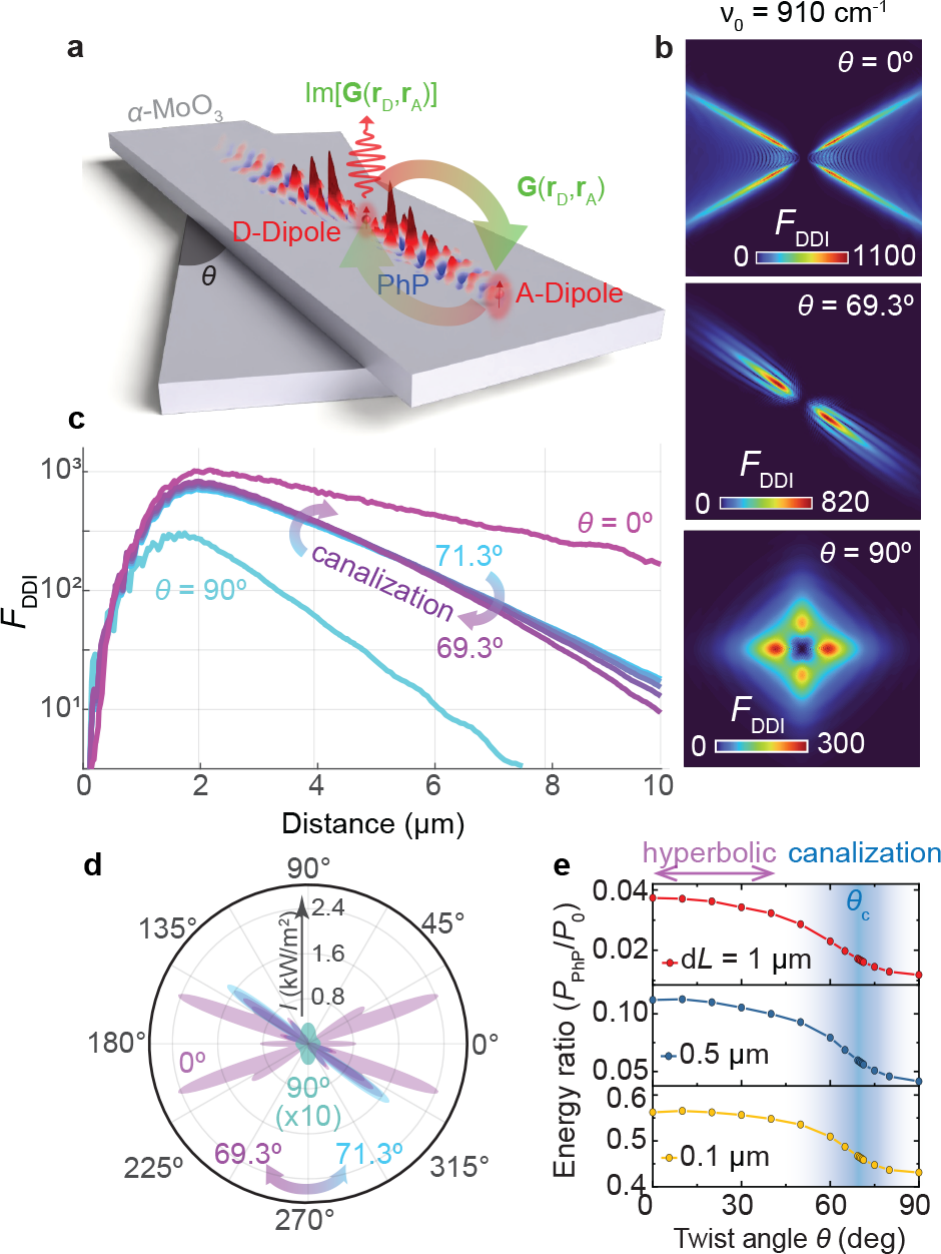
Long-range MIR DDIs mediated by PhPs in twisted bilayer α-MoO_3_. (a) Two dipoles on twisted α-MoO_3_ slabs at angle θ, interacting via PhPs described by the DGF **G̃**(**r**_*A*_, **r**_*D*_). (b) Normalized DDI enhancement *F*_DDI_ at 910 cm^–1^ for twist angles θ = 0°, 69.3°, and 90°. (c) *F*_DDI_ along the direction of maximum coupling (canalization or hyperbolic asymptotes) for the same system. (d) Real-space PhP intensity patterns as a function of twist angle, obtained analytically from the DGF. (e) Ratio of PhP power *P*_PhP_ to total dipole decay *P*_0_ versus twist angle for several distances from the donor.

At θ = 0° (Figure 4b, top), the enhancement pattern resembles that of the 200 nm single slab, with additional fine fringes near the origin from high-order modes.^68^ Quantitative differences arise because the previous case used a 2D model, whereas here we carry out a full 3D simulation. The interaction lengths are *L*_*p*_ = 3.5 μm along the hyperbola’s axis and *L*_*p*_ = 2.5 μm along its asymptotes, as derived from the analytical dispersion^65^ (see Supplementary Section VII). At θ = 69.3° (Figure 4b, middle), PhPs become canalized, propagating more directionally but with lower intensity and shorter interaction length (*L*_*p*_ = 1.96 μm). This trend intensifies at θ = 90° (Figure 4b, bottom), where propagation occurs in all directions with reduced range (*L*_*p*_ = 1 μm) and slightly asymmetric modes due to the broken symmetry between the layers. We note here that, in the twisted bilayer case, and more generally in twisted systems, or in systems where there is coupling between an anisotropic slab and a polaritonic substrate/superstrate,^76,77^ canalization emerges from the hybridization of polaritons supported by the top and bottom layers. This hybridization is inherently three-dimensional in nature: out-of-plane propagation plays an essential role, and the *z* components of the electromagnetic fields cannot be neglected. A full three-dimensional treatment is therefore required to correctly capture the physics.

Figure 4c shows *F*_DDI_ versus distance for different θ. The hyperbolic case (θ = 0°) yields the largest enhancement (*F*_DDI_ ≈ 1100) and longest range, while at θ = 90° the enhancement drops to ≈ 300. Near the so-called *magic angle* (θ ≈ 69–71°), the enhancement is slightly lower than in the hyperbolic case at all distances, despite the increased directionality. This behavior is consistent with Figure 4b, where canalization produces the most directional but not the strongest DDI patterns. The analytical polar plots of the PhP intensity maps at 5*L*_*p*_ (Figure 4d) further confirm this (see Supplementary Section VIII for the derivation), showing the balance between extremely directional interaction and long-range DDIs as a function of the twist angle, which provides a tuning knob for controlling MIR energy transfer.

To understand why canalized PhPs do not yield the strongest long-range DDIs, we analyze the dipole’s energy distribution among three decay channels: (1) guided PhPs, (2) negligible free-space radiation, and (3) localized absorption (quenching). Since rigorous near-to-far-field transformations are challenging in anisotropic media,^78^ we estimate the PhP power *P*_PhP_ from the Poynting vector’s surface integral over a cylindrical shell of radius *dL* and height 800 nm, excluding top and bottom surfaces to isolate in-plane energy flow. The ratio *P*_PhP_/*P*_0_, where *P*_0_ is the total dipole decay power, measures the in-coupling efficiency of PhPs (Figure 4e). For all distances, *P*_PhP_/*P*_0_ decreases as θ increases, with a sharp drop near the canalization (magic) angle θ_*c*_, explaining the trade-off between directionality and coupling strength. As distance grows, the ratio further declines due to optical losses. Since LDOS reflects available photonic states rather than coupling efficiency, the reduced *P*_PhP_/*P*_0_ near θ_*c*_ arises from the lower LDOS, not weaker coupling.

Therefore, canalization enhances the propagation directionality but inherently reduces the LDOS and in-coupling efficiency. In hyperbolic slabs (*ε*_*x*_ *ε*_*y*_ < 0), the interaction potential |*V*_dd_| diverges along the IFC asymptotes, producing super-Coulombic enhancement. However, canalized PhPs lack such divergence, yielding smaller |*V*_dd_|. This provides a quantitative explanation of how free-space radiation couples into PhP canalization, an insight crucial for future directional nanophotonics applications.^79^

In conclusion, we have provided a theoretical framework to describe long-range DDIs in strongly anisotropic media. Using single and twisted α-MoO_3_ slabs as representative strongly anisotropic materials, we have shown that low-loss hyperbolic PhPs can mediate exceptionally strong and long-range DDIs in the MIR, with extreme directionality, surpassing traditional MIR platforms, SPPs on gold and SPhPs in SiC, by more than 3 orders of magnitude, as well as those achieved by graphene plasmons. The coupling range can extend to beyond 50 μm (about five free-space wavelengths). We have also shown that canalization enhances directionality by confining propagation to a single in-plane direction, but this restriction inherently limits the in-coupling efficiency due to the reduced density of accessible states. Our study thus establishes hyperbolic PhPs as a promising platform for strong, long-range MIR DDIs and provides a quantitative framework for understanding the coupling of free-space radiation into canalized PhPs, highlighting the interplay between confinement, long-range interactions and directionality. These insights open avenues for directional vibrational energy transport, thermal management, nanoscale sensing, and MIR quantum information technologies. Our framework shows that HPhPs can mediate strong long-range DDIs and energy transfer in the MIR, and could be extended to other frequency ranges, leveraging the expanding class of in-plane hyperbolic materials and their twisted structures, supporting hyperbolic plasmon polaritons in the visible,^45,46^ hyperbolic plasmon, exciton, and phonon polaritons in the near- and mid-infrared,^36,55,80^ and hyperbolic phonon polaritons in the far-infrared.^81^

## Supplementary Material



## Notes

Permittivity of α-MoO_3_ in the frequency range 800–920 cm^–1^; comparison of the dipole–dipole interaction energy enhancement *F*_DDI_ for a single slab: analytical DGF vs full-wave simulations; real and imaginary parts of the wavevector *k* as a function of the in-plane angle φ; propagation length to polariton wavelength ratio in a single α-MoO_3_ slab; FoM for enhancement and long-range propagation via Green-function formalism; estimates of net energy transfer rates and efficiencies using representative mid-infrared emitters; calculation of the propagation length of PhPs in twisted α-MoO_3_ slabs; PhP electric field distribution in a twisted structure of biaxial slabs based on the dyadic Green’s function (PDF)
