# Supplementary material for: Long-Range Mid-Infrared Energy Transfer Mediated by Hyperbolic Phonon Polaritons
Source: Nano Lett. 2026 May 28;26(24):7910–7. doi: 10.1021/acs.nanolett.6c01042 (PMC13307272; doi:10.1021/acs.nanolett.6c01042)
Supplement: Supplementary file 1 [file nl6c01042_si_001.pdf]

SUPPORTING INFORMATION FOR
Long-range mid-infrared energy transfer mediated by hyperbolic phonon polaritons

Gonzalo Álvarez-Pérez [1,*] Simone De Liberato [2,3] and Huatian Hu [1,*]

[1] *Istituto Italiano di Tecnologia, Center for Biomolecular Nanotechnologies, Via Barsanti 14, 73010 Arnesano, Italy*
[2] *Istituto di Fotonica e Nanotecnologie, Consiglio Nazionale delle Ricerche (CNR), Piazza Leonardo da Vinci 32, Milano, 20133, Italy*
[3] *School of Physics and Astronomy, University of Southampton, Southampton SO17 1BJ, United Kingdom*

(Dated: May 16, 2026)

CONTENTS

I. Permittivity of α-MoO$_3$ in the frequency range 800-920 cm^{-1} 1

II. Comparison of the dipole–dipole interaction energy enhancement F_{DDI} for a single slab: analytical DGF vs full-wave simulations 1

III. Real and imaginary parts of the wavevector k as a function of the in-plane angle φ 2

IV. Propagation length to polariton wavelength ratio in a single α-MoO$_3$ slab 2

V. FoM for enhancement and long-range propagation via Green-function formalism 3

VI. Estimates of net energy transfer rates and efficiencies using representative mid-infrared emitters 4
- A. Finite linewidths and spectral overlap 4
- B. Detuning 7
- C. Dephasing 8
- D. Nonradiative decay and transfer efficiency 8

VII. Calculation of the propagation length of PhPs in twisted α-MoO$_3$ slabs 8

VIII. PhP electric field distribution in a twisted structure of biaxial slabs based on the dyadic Green's function 8

References 9

I. PERMITTIVITY OF α-MOO$_3$ IN THE FREQUENCY RANGE 800-920 CM^{-1}

Figure 1 shows the real and imaginary parts of the in-plane permittivity components of α-MoO$_3$, ε_x and ε_y, in the spectral range of interest, from 800 to 920 cm^{-1}. For visibility, both the real and imaginary parts of ε_y have been multiplied by 100. The RB associated with the phonon along the x axis starts around 820 cm^{-1}, while ε_y remains small and crosses zero near 850 cm^{-1}, in agreement with values reported in the literature [1]. The permittivity values used for both analytical calculations and full-wave numerical simulations in this work.

II. COMPARISON OF THE DIPOLE–DIPOLE INTERACTION ENERGY ENHANCEMENT F_{DDI} FOR A SINGLE SLAB: ANALYTICAL DGF VS FULL-WAVE SIMULATIONS

Figure 2 compares the single-slab analytical model with full-wave numerical simulations for a 200 nm α-MoO$_3$ slab. Specifically, we evaluate the dipole–dipole interaction (DDI) enhancement factor F_{DDI} at the frequency where it reaches its maximum, approximately $900\ \mathrm{cm}^{-1}$, and observe excellent agreement between the two approaches. The two panels show the spatial distribution of the DDI enhancement at 20 nm above the slab. Both plots reproduce the characteristic hyperbolic PhP pattern, with key features—including the spatial extent of the enhanced region ($\sim 50\ \mu$m), the opening angle of the hyperbolic sector, and

* gonzalo.alvarezperez@iit.it
* huatian.hu@iit.it

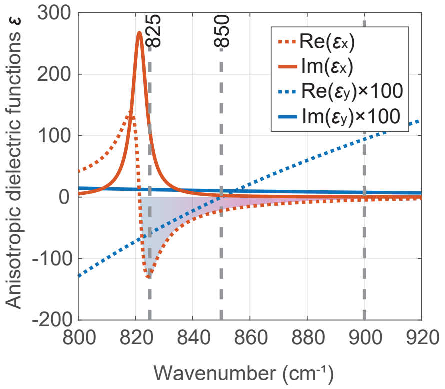

Figure 1. **Dielectric funtions of α-MoO₃.** The real (dashed lines) and imaginary (solid lines) part of x-component (red) and y-component (blue) of the permittivity.

the peak enhancement values (reaching ~ 4200 along the two symmetric hyperbolic asymptote directions on the shared color scale).

We emphasize that the 2D analytical model is not only significantly more computationally efficient, but also provides a closed-form expression for the dyadic Green's function (DGF), offering direct physical insight into the origin of the enhancement. In particular, it reveals that the enhancement arises from a divergence in the photonic density of states along the hyperbolic asymptote directions. However, extending this analytical framework to the twisted bilayer case is considerably more involved. For this reason, we employ full 3D finite-element method (FEM) simulations in the twisted-bilayer section.

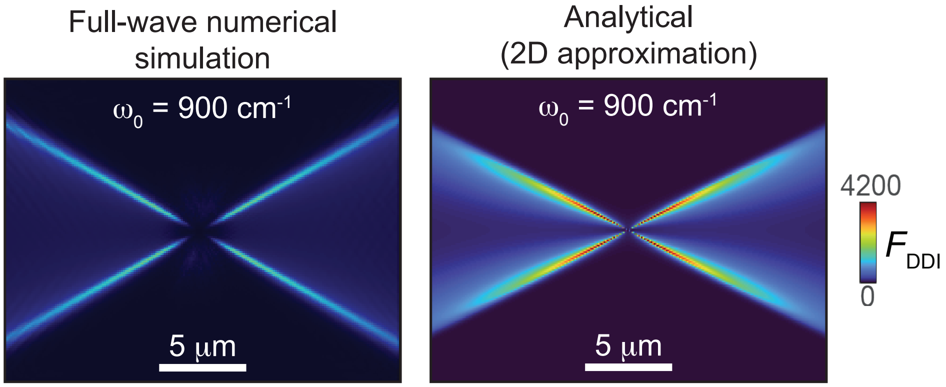

Figure 2. **Comparison of the full-wave numerical (left) and analytical (right) DDI enhancement factor F_{DDI}, shown as spatial maps in the plane of a 200 nm α-MoO$_3$ slab at $\omega_0 = 900\,\mathrm{cm}^{-1}$.** Both panels reproduce the characteristic hyperbolic pattern, with strong enhancement concentrated along the asymptote directions. The color scale (0–4200) is shared between the two panels. The scale bar corresponds to 5 μm.

III. REAL AND IMAGINARY PARTS OF THE WAVEVECTOR k AS A FUNCTION OF THE IN-PLANE ANGLE φ

In this Section we analytically calculate the real and imaginary parts of the wavevector k as a function of the in-plane angle φ for PhPs in a single 200-nm-thick α-MoO$_3$ slab at $\nu_0 = 900$ cm$^{-\circ}$. The result is shown in Fig. 3.

IV. PROPAGATION LENGTH TO POLARITON WAVELENGTH RATIO IN A SINGLE α-MOO$_3$ SLAB

In this Section we analytically calculate the ratio between the propagation length and wavelength of PhPs in a single α-MoO$_3$ slab: $L_p/\lambda_p = \mathrm{Re}(k)/2\pi\mathrm{Im}(k)$. The result is shown in Fig. 4, showing a maximum at ≈ 870 cm^{-1}.

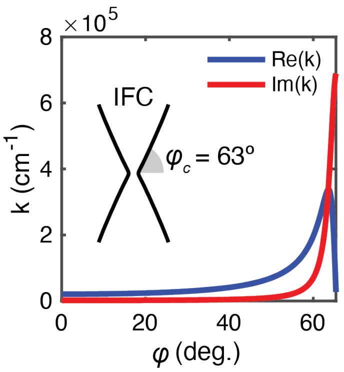

Figure 3. **Real and imaginary parts of the wavevector k as a function of the in-plane angle φ for PhPs in a single 200-nm-thick α-MoO$_3$ slab.**

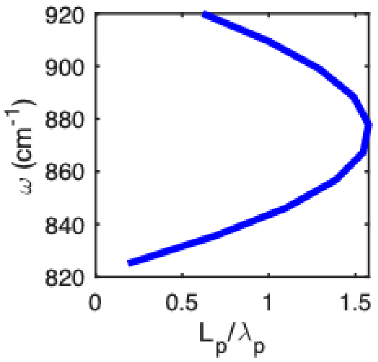

Figure 4. **Propagation length to polariton wavelength ratio in a single α-MoO$_3$ slab.**

V. FOM FOR ENHANCEMENT AND LONG-RANGE PROPAGATION VIA GREEN-FUNCTION FORMALISM

We can rewrite our DDI potential energy enhancement in polar coordinates as

$$F_{\mathrm{DDI}}(R,\theta,\omega) = \frac{\left|\tilde{G}_{zz}(R,\theta,\omega)\right|}{\left|\tilde{G}^0_{zz}(R,\omega)\right|}, \tag{1}$$

where $\tilde{G}_{zz}$ is the zz component of the dyadic Green's function (DGF) in the 2D anisotropic layer and $\tilde{G}^0_{zz}$ is its free-space counterpart.

For planar systems, the DGF can be expressed through the Sommerfeld–Weyl expansion, which decomposes the field radiated by a dipole into a continuum of plane waves with in-plane wavevector $\mathbf{k}_\parallel = (k_x, k_y)$. For a p-polarized source, only the p-polarized component contributes to the zz field, leading to [2, 3]

$$\hat{G}_{pp}(\mathbf{r},\mathbf{r}') = \frac{i}{8\pi^2}\int \frac{d^2\mathbf{k}_\parallel}{k_{1z}}\, e^{i\mathbf{k}_\parallel\cdot\Delta\mathbf{r}_\parallel}\, T^{pp}(\mathbf{k}_\parallel,\omega)\, e^{i(k_{1z}z'-k_{2z}z)}, \tag{2}$$

where $k_{iz} = \sqrt{k_0^2\varepsilon_i - k_\parallel^2}$ are the out-of-plane wavevector components in media $i = 1, 2$, $\Delta\mathbf{r}_\parallel$ is the in-plane separation vector, and T^{pp} is the Fresnel transmission coefficient for p polarization. Equation (2) is the DGF used in thin biaxial layers [2].

When the structure supports a surface polariton, T^{pp} exhibits a pole at the complex in-plane wavevector $\mathbf{k}_s = (k_x, k_y)$ that satisfies the dispersion relation $D_p(\mathbf{k}_s,\omega) = 0$. In the limit of large in-plane separation $R = |\mathbf{R}|$, the field is dominated by the residue of this pole, yielding the asymptotic form

$$\tilde{G}^{(\mathrm{surf})}_{zz}(R,\theta;\omega) \simeq A(\theta,\omega)\,\frac{e^{i\mathbf{k}_s\cdot\mathbf{R}}}{\sqrt{R}}, \tag{3}$$

where the factor $1/\sqrt{R}$ originates from the two-dimensional cylindrical spreading of the polaritonic wave. Writing $\mathbf{R} = R(\cos\theta, \sin\theta)$ and $\mathbf{k}_s = (k_x, k_y)$ gives $\mathbf{k}_s\cdot\mathbf{R} = (k_x\cos\theta + k_y\sin\theta)R \equiv k(\theta)R$. Because the mode is generally lossy

and anisotropic, $k(\theta) = k'(\theta) + ik''(\theta)$ is complex, and the field amplitude decays as

$$\tilde{G}_{zz}^{(\mathrm{surf})}(R, \theta; \omega) \propto \frac{e^{i\,\mathrm{Re}k(\theta)\,R}}{\sqrt{R}}\, e^{-R/L_p(\theta,\omega)}, \qquad L_p(\theta, \omega) = \frac{1}{\mathrm{Im}\,k(\theta, \omega)}. \tag{4}$$

Substituting Eq. (4) into the definition of F_{DDI}, and noting that the vacuum Green function exhibits the same lateral $1/\sqrt{R}$ dependence, one obtains the asymptotic form

$$F_{\mathrm{DDI}}(R, \theta, \omega) \simeq F_{\mathrm{DDI}}(0, \theta, \omega)\, \exp[-\,R/L_p(\theta, \omega)]. \tag{5}$$

Equation (5) demonstrates that the DDI enhancement inherently encapsulates two key characteristics of the platform: (i) the local enhancement of the DDI potential energy $F_{\mathrm{DDI}}(0, \theta, \omega)$, which includes the Purcell-like enhancement in its imaginary part and the Lamb-shift contribution in its real part; and (ii) the field propagation length $L_p(\theta, \omega) = 1/\mathrm{Im}\{k(\theta, \omega)\}$, which quantifies the range of polariton-mediated interactions along the direction θ. Hence, $F_{\mathrm{DDI}}(R, \theta, \omega)$ itself acts as a comprehensive figure of merit, simultaneously combining enhancement and range in a single observable.

Figure 5 shows the integral

$$\int dRF_{\mathrm{DDI}}(R, \theta, \omega) \simeq \int dRF_{\mathrm{DDI}}(0, \theta, \omega)\, \exp[-\,R/L_p(\theta, \omega)]$$

as a function of ν, which shows a maximum at 850 cm^{-1}.

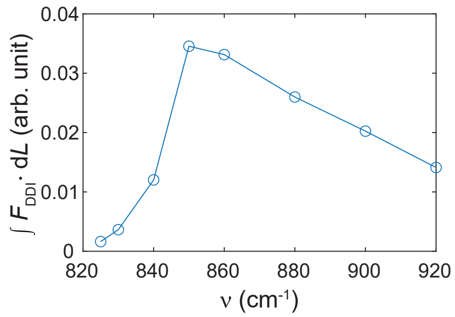

Figure 5. **Integral $\int dRF_{\mathrm{DDI}}(R, \theta, \omega) \simeq \int dRF_{\mathrm{DDI}}(0, \theta, \omega)\, \exp[\,R/L_p(\theta, \omega)]$ as a function of ν.** The curve shows a maximum at 850 cm^{-1}

VI. ESTIMATES OF NET ENERGY TRANSFER RATES AND EFFICIENCIES USING REPRESENTATIVE MID-INFRARED EMITTERS

In the present work, we focus on the mid-infrared regime, where dipoles are associated with vibrational or thermal excitations. In this context, the donor–acceptor description remains an ideal but physically meaningful framework. To assess our proposal under realistic conditions, we have performed order-of-magnitude estimates of net energy transfer rates and efficiencies using representative mid-infrared emitters, including finite linewidths, detuning, dephasing, nonradiative decay, and spectral overlap. This is done for a single slab within our 2D approximation, which can be evaluated analytically and represents the optimal case in which the F_{DDI} is maximized. For the twisted configuration, we also outline the corresponding formalism, which is equivalent except for the modification of the Green's function, but do not evaluate it explicitly.

A. Finite linewidths and spectral overlap

The energy transfer rate from a donor to an acceptor, normalized by the vacuum spontaneous decay rate γ_0, is given by [3]:

$$\frac{\gamma_{D\to A}}{\gamma_0} = \frac{9c^4}{8\pi R^6} \int \frac{f_D(\omega)\,\sigma_A(\omega)}{n^4\omega^4}\, T_{\mathrm{ET}}(\omega) \cdot d\omega, \tag{6}$$

where the function that determines the strength of the energy transfer T_{ET} reads

$$T_{\mathrm{ET}}(\omega) = 16\pi^2 k^4 R^6 \left|G_{zz}(\omega)\right|^2 . \tag{7}$$

As justified in the manuscript, we assume that both donor and acceptor dipoles are oriented along the vertical axis (z-axis), so that the term $\hat{n}_A \cdot \overleftrightarrow{G} \cdot \hat{n}_D$ reduces to the G_{zz} component (proportional to V_{dd}). In the equation above, c is the speed of light, R is the donor–acceptor separation, $f_D(\omega)$ is the spectral density of the donor, $\sigma_A(\omega)$ is the absorption cross-section of the acceptor (both assumed to have Lorentzian lineshapes), n is the refractive index, ω the angular frequency, and k is the wavenumber. The DDI enhancement of energy transfer between realistic emitters is then quantified by the ratio of overlap integrals:

$$\frac{\gamma_{D\to A}}{\gamma^0_{D\to A}} = \frac{\displaystyle\int f_D(\omega)\,\sigma_A(\omega)\,\left|G_{zz}(\omega,\Delta\mathbf{r})\right|^2 d\omega}{\displaystyle\int f_D(\omega)\,\sigma_A(\omega)\,\left|G^0_{zz}(\omega,\Delta\mathbf{r})\right|^2 d\omega}. \tag{8}$$

We first compute the frequency-dependent Green's function $G_{zz}(\omega, \Delta\mathbf{r})$. Crucially, unlike the isotropic case, where G_{zz} depends only on $|\Delta\mathbf{r}|$ due to azimuthal symmetry, the Green's function here depends explicitly on in-plane angle owing to the anisotropy of the system (Fig. 6). Fig. 6 shows $|G_{zz}(\omega, |\Delta\mathbf{r}| = 15\,\mu\mathrm{m})|$ as a representative example. Both G_{zz} and V_{dd} exhibit fourfold symmetric maxima over the full azimuthal range of 2π. In Fig. 7, we evaluate the frequency-dependent $G_{zz}(\omega, \Delta\mathbf{r})$ at a fixed distance from the source. We choose a distance $|\Delta\mathbf{r}| = 15\,\mu\mathrm{m}$, corresponding to approximately $1.5\lambda_0$, in order to probe the long-range response; this choice is otherwise arbitrary. At this radius, we select the in-plane direction along which $|G_{zz}(\omega_0, \Delta\mathbf{r})|$ is maximized for a given frequency ω_0. As an example, to obtain the red curve in Fig. 7, we first determine the direction corresponding to $\Delta\mathbf{r} = (x_0, y_0)$ that maximizes $|G_{zz}(\omega_0 = 850\,\mathrm{cm}^{-1},\ |\Delta\mathbf{r}| = 15\,\mu\mathrm{m})|$. We then compute the electric field at this fixed direction over a range of frequencies, yielding $G_{zz}(\omega, \Delta\mathbf{r})$, and thus $|G_{zz}(\omega)|$ at that point, as shown in Fig. 7. For isotropic materials, this procedure is straightforward, since all directions at a fixed distance from the dipole exhibit identical optical responses. In contrast, for anisotropic materials, the response depends on the isofrequency contour, leading to a nontrivial angular dependence.

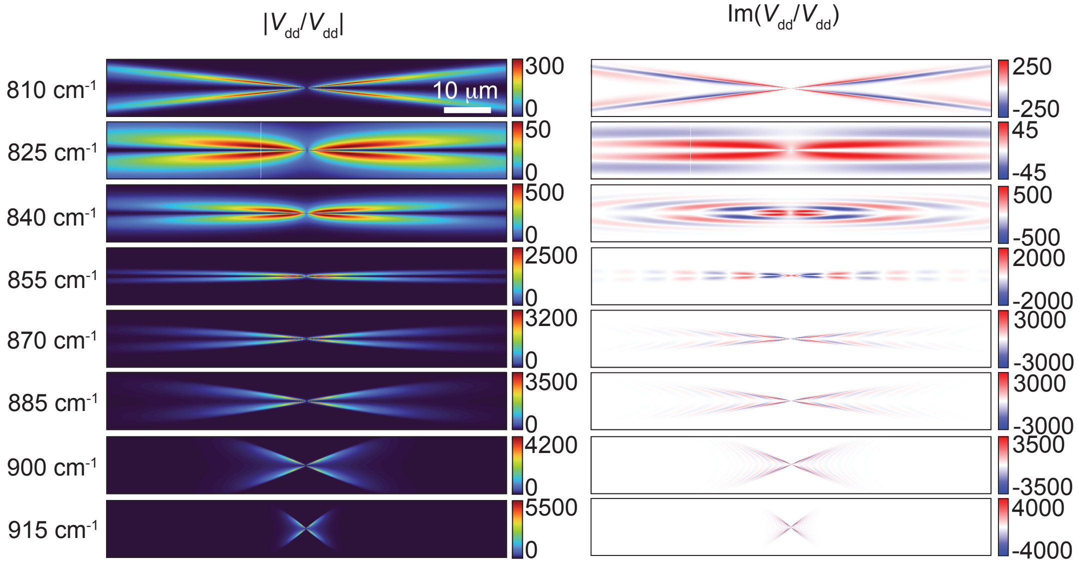

Figure 6. **Dipole-dipole interaction potential map at different frequencies.** The hyperbolic PhP-engineered Green's function at a specific location; the spectral distribution (linewidth) of the emitter may not couple to the PhP-engineered vacuum field with the same efficiency.

Notably, at $850\,\mathrm{cm}^{-1}$ — where canalization occurs due to a minimal opening angle — only a single spectral peak appears, since no other PhP trajectory passes through the same point. In contrast, the cases at 825 and $900\,\mathrm{cm}^{-1}$, which have larger opening angles, exhibit multiple peaks arising from PhP contributions at different wavelengths along nearby trajectories. This is a direct consequence of the hyperbolic nature of the system and has no counterpart in isotropic platforms such as metallic plasmons, graphene plasmons, or conventional waveguides. In those systems, resonance-like spectral features are typically associated with localized polaritons in cavities, where the in-plane wavevector is discretized. The emergence of such features in a flat, open slab is therefore a distinctive property of hyperbolic media. By contrast, the vacuum G_{zz} (isotropic) shows a flat, featureless tail without resonant features, as shown in the lower panel of Fig. 7.

In order to provide an estimate with a real emitter, we consider a vibrational mode of the C–H bond at $850\,\mathrm{cm}^{-1}$ with approximately $10\,\mathrm{cm}^{-1}$ linewidth [4, 5]. Note that the intrinsic linewidth of a vibrational mode can be 3–$5\,\mathrm{cm}^{-1}$ [6]; we adopt the higher value to represent a more conservative scenario. This linewidth is comparable to that of the Green's function, meaning that the spectral density of the emitter will cause the overlap integral to deviate from the idealized single-dipole result. We consider the on-resonance case in which donor and acceptor are identical species separated by a fixed distance. Evaluating Eq. (8) yields $\gamma_{D\to A}/\gamma^0_{D\to A} = 1.196 \times 10^7$, which corresponds to a correction factor of 0.738 relative to the ideal ratio $|G_{zz}|^2/|G^0_{zz}|^2 = |V_{dd}|^2/|V^0_{dd}|^2$. In the idealized limit of a spectrally infinitesimal dipole (FWHM $\to 0$), the two ratios coincide exactly.

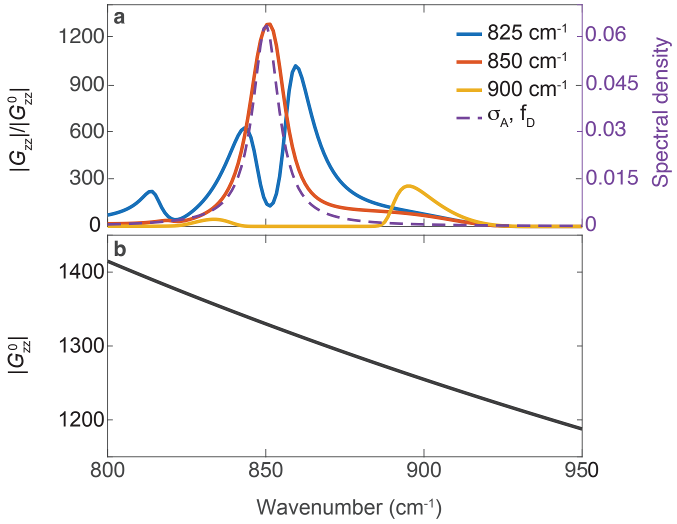

Figure 7. **Absolute value of the dyadic Green's function at the location of its maximum for $825\,\mathrm{cm}^{-1}$ (blue), $850\,\mathrm{cm}^{-1}$ (red), and $900\,\mathrm{cm}^{-1}$ (yellow).** The background shows the spectral density of the donor $f_D(\omega)$ and acceptor $\sigma_A(\omega)$ entering the overlap integral. The gray solid line shows the vacuum Green's function at $|\Delta\mathbf{r}| = 15\,\mu\mathrm{m}$, which is azimuthal-independent due to isotropy and symmetry, stressing the uniqueness of the resonance-like features arising from hyperbolic and canalized PhPs.

Analogous calculations performed for vibrational modes centered at 825 and $900\,\mathrm{cm}^{-1}$ yield correction factors of 2.6 and 0.9 relative to $|V_{dd}|^2/|V^0_{dd}|^2$, with ideal enhancement values of 5.03×10^3 and 4.51×10^4, respectively. The correction factor exceeding unity at $825\,\mathrm{cm}^{-1}$ warrants particular attention: at this frequency, ray canalization is a loss-dominated process that produces only weak enhancement. The Green's function therefore exhibits a local minimum (dip) near $825\,\mathrm{cm}^{-1}$ (Fig. 7), and a spectrally broader emitter centered at this frequency can capture additional enhancement from neighboring frequencies where the PhP-mediated coupling is stronger.

As shown in Fig. 8, the correction ratio map for a Lorentzian emitter at $850\,\mathrm{cm}^{-1}$ with $10\,\mathrm{cm}^{-1}$ FWHM demonstrates that $|G_{zz}|^2/|G^0_{zz}|^2$ (equivalently, $|V_{dd}|^2/|V^0_{dd}|^2$) reproduces the full overlap integral $\gamma_{D\to A}/\gamma^0_{D\to A}$ across the map, with a correction ratio close to unity throughout. This confirms that the ideal dipole approximation provides a reliable estimate of the PhP-enhanced energy transfer rate for emitters with realistic spectral linewidths.

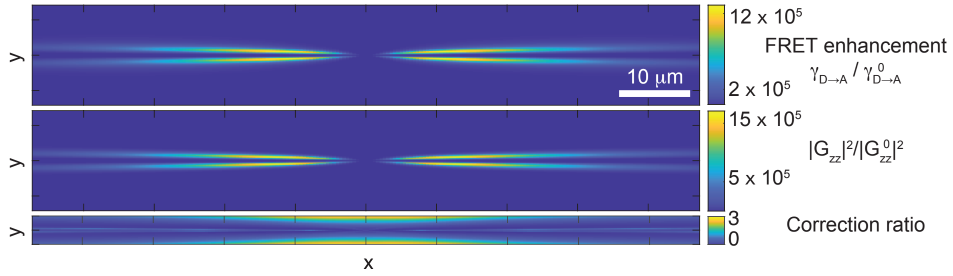

Figure 8. **Comparison of the FRET enhancement map for a Lorentzian emitter at $850\,\mathrm{cm}^{-1}$ with $10\,\mathrm{cm}^{-1}$ linewidth.** Upper panel: full overlap integral $\gamma_{D\to A}/\gamma^0_{D\to A}$. Middle panel: ideal dipole ratio $|G_{zz}|^2/|G^0_{zz}|^2$ (equivalently $|V_{dd}|^2/|V^0_{dd}|^2$). Lower panel: correction ratio $\left(\gamma_{D\to A}/\gamma^0_{D\to A}\right) / \left(|G_{zz}|^2/|G^0_{zz}|^2\right)$. The map is restricted to the region near the PhP propagation direction; the large values near $|y| \to \infty$ arise from numerical error due to a vanishingly small denominator.

B. Detuning

Detuning is an important factor in the overlap integral as it determines how much energy can be effectively transferred to the acceptor. Adopting a linewidth of $\mathrm{FWHM} = 10\,\mathrm{cm}^{-1}$ as above, we consider detuning between acceptor and donor resonances ranging from -4 to 4 times the FWHM. As shown in the upper panel of Fig. 9, we illustrate one specific case with $\delta = -2\,\mathrm{FWHM}$. In this context, the lower panel shows the correction that must be made from the on-resonance case if a detuning is introduced. We see that, if the detuning reaches FWHM, the FRET rate drops to 28.5%. It will be further decreased to 7.8% if 2FWHM is reached. They are all compared with the on-resonance FRET rate $\gamma^{\mathrm{res}}_{D\to A}$.

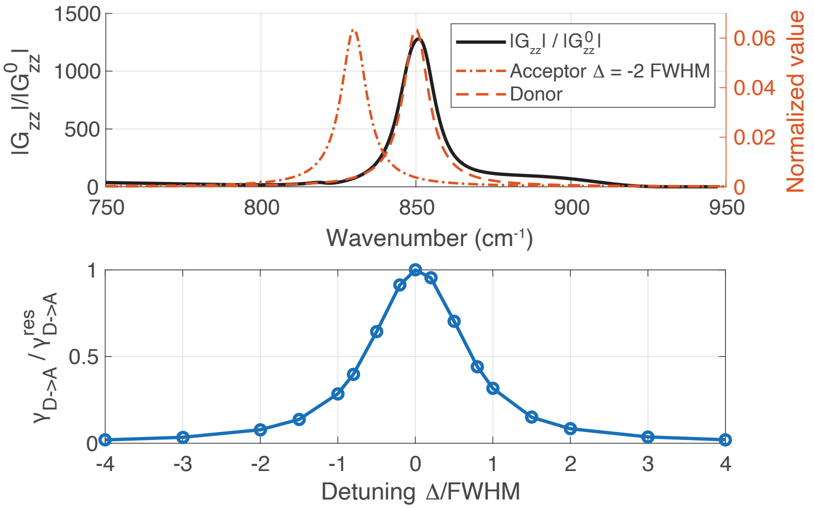

Figure 9. **Energy transfer rate versus donor–acceptor detuning.** Top: the squared field amplitude $|G_{zz}|^2/|G^0_{zz}|^2$ (black) compared with the normalized donor (orange, dashed) and acceptor (orange, dash-dotted) spectra, showing spectral overlap near resonance. Bottom: the normalized transfer rate $\gamma_{D\to A}/\gamma^{\mathrm{res}}_{D\to A}$ as a function of detuning normalized to the FWHM, peaking at zero detuning and decreasing symmetrically away from resonance.

We further investigate the role of linewidth in the FRET rate (Fig. 10). The upper panel shows two examples with $\mathrm{FWHM}_0 = 10\,\mathrm{cm}^{-1}$ and $2\,\mathrm{FWHM}_0 = 20\,\mathrm{cm}^{-1}$. With a broader linewidth, the FRET rate decreases gradually: a 50% increase in FWHM results in a drop of the FRET rate to 55% of its value at FWHM_0.

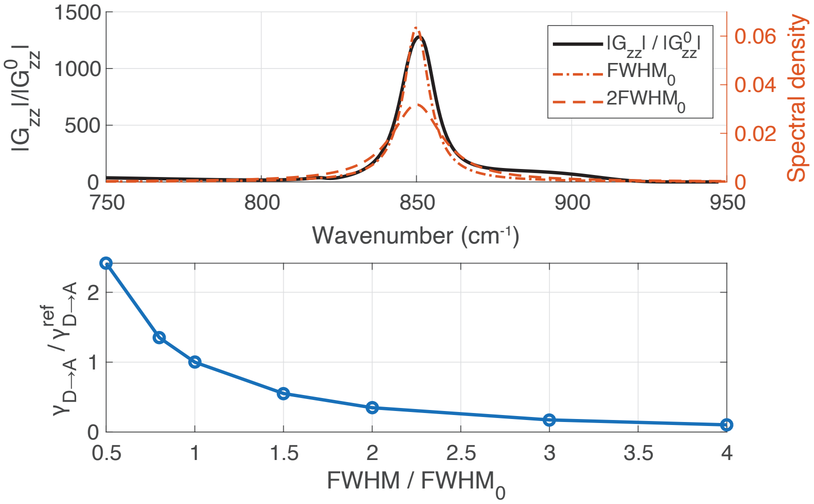

Figure 10. **Effect of spectral linewidth on MIR energy transfer.** Top: the normalized cavity response $|G_{zz}|^2/|G^0_{zz}|^2$ (black) compared with emitter spectra of varying linewidths (colored dashed/dotted curves labeled by FWHM ratios), showing increasing spectral broadening relative to the cavity. Bottom: the normalized transfer rate $\gamma_{D\to A}/\gamma^{\mathrm{res}}_{D\to A}$ versus the linewidth ratio $\mathrm{FWHM}/\mathrm{FWHM}_0$, illustrating a monotonic decrease in efficiency as the emitter linewidth broadens.

C. Dephasing

While dephasing is best described in a fully quantum mechanical approach, which is out of the scope of this work, its broadening can be phenomenologically captured by its impact on the linewidth, as shown in Fig. 10. Pure dephasing, characterized by a rate γ_φ, broadens the emitter linewidth beyond the population decay rate γ_1 according to $\gamma_{\text{total}} = \gamma_1/2 + \gamma_\varphi$. For MIR molecular vibrations at room temperature, pure dephasing is typically the dominant broadening mechanism, with $T_2^* \sim$ 1–2 ps [6], corresponding to homogeneous linewidths of $\sim$ 5–10 cm^{-1}. In our formalism, dephasing enters effectively as an additional broadening of the spectral lineshape. Since we already adopted a conservative linewidth of $10\,\text{cm}^{-1}$ — larger than the intrinsic T_1-limited value of 3–5 cm^{-1} — our estimates implicitly incorporate a significant dephasing contribution. The correction ratio of 0.738 at $850\,\text{cm}^{-1}$ therefore already reflects a realistic dephased emitter rather than an idealized two-level system.

D. Nonradiative decay and transfer efficiency

The nonradiative decay can be attributed to two distinct processes: (1) the internal quantum efficiency (IQE) of the quantum emitter, defined as the ratio of the radiative decay rate to the total decay rate — an intrinsic material property governed by defects and phonon scattering; and (2) the coupling of the emitter, modeled as an effective dipole, to the surrounding electromagnetic environment, fully encoded in the dyadic Green's function G_{zz}. This quantity captures the complete photonic local density of states, of which PhP-mediated coupling is the dominant contribution in our system. The external quantum efficiency can therefore be obtained by incorporating the IQE as a multiplicative correction factor.

The absolute transfer efficiency is

$$\eta \approx \frac{\gamma_{D\to A}}{\gamma_{D\to A} + \gamma_{nr}}, \tag{9}$$

valid in the limit $\gamma_{\text{rad}} \ll \gamma_{nr}$ typical of MIR vibrational modes, and neglecting back-transfer from acceptor to donor — an idealization that sets an upper bound on efficiency in the on-resonance, identical-species case considered here. The crossover distance R^*, defined by $\gamma_{D\to A}(R^*) = \gamma_{nr}$, determines the range over which efficient transfer is achievable. Writing $\gamma_{D\to A}(R) = F_{\text{DDI}}(R) \cdot \gamma^0_{D\to A}(R)$ and approximating the vacuum rate as $\gamma^0_{D\to A}(R) = A/R^6$ with $A \propto \mu^4$, the crossover condition becomes:

$$R^* = \left(\frac{AB}{\gamma_{nr}}\right)^{1/(6+\alpha)}, \tag{10}$$

where $F_{\text{DDI}}(R) \approx B \cdot R^{-\alpha}$ is a power-law fit to the numerically computed enhancement along the asymptote direction. From Fig. 3 (right panel), F_{DDI} at $850\,\text{cm}^{-1}$ maintains $\sim$500 at $10\,\mu\text{m}$ and $\sim$100 at $40\,\mu\text{m}$, yielding $\alpha \approx 1.16$ and $B \approx 7200\,\mu\text{m}^{1.16}$ — far slower than the vacuum near-field R^{-6} scaling. In vacuum ($B = 1$, $\alpha = 0$), R^* reduces to $(A/\gamma_{nr})^{1/6}$. The ratio $R^*_{\text{PhP}}/R^*_{\text{vac}}$ then quantifies the extension of the transfer range in α-MoO_3.

For a representative C–H mode ($\mu \sim 0.1\,\text{D}$, $\gamma_{nr} \sim 1\,\text{ps}^{-1}$ [4]), $A \sim 10^{-4}\,\mu\text{m}^6 \cdot \text{ps}^{-1}$, giving $R^*_{\text{vac}} \sim 70\,\text{nm}$ and $R^*_{\text{PhP}} \sim 1\,\mu\text{m}$ — a tenfold extension in distance, or three orders of magnitude in volume. All competing MIR platforms (Au SPP, SiC, graphene) fall below $F_{\text{DDI}} \sim 1$ before $R = 30\,\mu\text{m}$ (Fig. 3, left panel, note the $\times 100$ rescaling), placing them in the $\gamma_{D\to A} \ll \gamma_{nr}$ regime and leaving their R^* below $1\,\mu\text{m}$. For stronger MIR emitters such as intersubband transitions ($\mu \sim$ 1–10 D), where $R^*_{\text{PhP}} \propto \mu^{4/(6+\alpha)}$, R^* scales as $\mu^{0.56}$ and shifts into the micron-to-tens-of-microns range: for $\mu = 10\,\text{D}$, $R^*_{\text{PhP}} \sim 14\,\mu\text{m}$, and $\eta \to 1$ becomes achievable at distances beyond the reach of, to our knowledge, any established MIR platform.

VII. CALCULATION OF THE PROPAGATION LENGTH OF PHPS IN TWISTED α-MOO$_3$ SLABS

The propagation length values L_p of PhPs in twisted α-MoO_3 slabs, as reported in the main text, were calculated using $L_p = 1/\text{Im}(k)$, where k is the wavevector along the direction of interest. This wavevector was obtained from the analytical dispersion relation for twisted trilayers, given by Eq. (S85) in the Supplementary Information of Ref. [7], with the thickness of the top layer set to zero.

VIII. PHP ELECTRIC FIELD DISTRIBUTION IN A TWISTED STRUCTURE OF BIAXIAL SLABS BASED ON THE DYADIC GREEN'S FUNCTION

To calculate the electric field distribution generated by a point source on top of a twisted structure of biaxial slabs, we employ the dyadic Green's function (DGF) formalism, which provides a solution to the vectorial Maxwell's equations for a dipolar

source. The DGF, $\tilde{\mathbf{G}}(\mathbf{r}_A, \mathbf{r}_D)$, represents the electric field at point $\mathbf{r}_A$ produced by a point source located at $\mathbf{r}_D$. The total electric field generated by a point dipole with an electric dipole moment $\boldsymbol{\mu}$ can be computed at any in-plane distance between the donor and acceptor dipoles $\mathbf{r}_A - \mathbf{r}_D$ above the top slab using the expression [3]:

$$\mathbf{E}(\mathbf{r}_A, \mathbf{r}_D) = 4\pi k_0^2 \tilde{\mathbf{G}}(\mathbf{r}_A, \mathbf{r}_D)\boldsymbol{\mu}.$$

As discussed in the main text, for our analysis, we restrict our consideration to a vertically polarized electric point dipole $\boldsymbol{\mu} = (0, 0, \mu)^T$ and focus on the z-component of the electric field. We further simplify the problem by considering only the p-polarized contribution to the DGF, denoted as G_{pp}, which describes the p-polarized field component excited by a p-polarized point source. The explicit form of this DGF can be found in refs. [3, 7], allowing us to express the polariton electric field (specifically, its z-component) as:

$$E_z(\mathbf{r}_A) = \frac{ik_0^3\mu}{2\pi\varepsilon_1} \int \frac{k_\parallel^2\, d^2\mathbf{k}_\parallel}{k_0^3 k_{1z}} \left(e^{ik_{1z}(z-z')} + r^{\mathrm{pp}} e^{ik_{1z}(z+z')}\right) e^{i\mathbf{k}_\parallel \cdot \mathbf{r}_\parallel}. \tag{11}$$

where r^{pp} is the amplitude of the Fresnel's reflection coefficient for the structure, $k_{1z} = \sqrt{k_0^2\varepsilon_1 - k_\parallel^2}$ stays for the z component of the wavevector in the isotropic superstrate (with dielectric permittivity $\varepsilon_1 = 1$), $\mathbf{r}_\parallel$ is the in-plane position vector and $k_\parallel$ is the in-plane component of the wavevector. Here, we have set the in-plane component of the donor dipole position vector to zero ($\mathbf{r}_D = 0$).

Assuming the polariton contribution dominates over other field components, we simplify Eq. (11) by neglecting the non-singular first term in the integrand, as the polariton contribution arises from the poles of the Fresnel coefficient r^{pp}. Additionally, since polaritons exhibit large wavevectors compared to free-space propagating waves (in the superstrate), we apply a large-k approximation, where $k_{1z} = ik_\parallel = iqk_0$ and $q = k_\parallel/k_0$ is the normalized in-plane wavevector. Converting the integral to polar coordinates, $(q_x, q_y) \to (q, \alpha_q)$ and $(x, y) \to (\rho, \alpha_\rho)$, we obtain the final expression for the electric field:

$$E_z(\mathbf{r}_A, \alpha_\rho) = E_0 \int_0^\infty \mathrm{d}q \int_0^{2\pi} \mathrm{d}\alpha_q\, q^2 r^{pp}(q, \alpha_q) e^{-qk_0(z+z')} e^{iqk_0\rho\cos(\alpha_q - \alpha_\rho)}, \tag{12}$$

where $E_0 = (k_0^3\mu)/(2\pi\varepsilon_1)$ is a constant prefactor. This expression allows us to compute the electric field as a function of the in-plane angle α_ρ, and by squaring it, we obtain the intensity plots presented in Fig. 3 of the main text.

REFERENCES

[1] G. Álvarez Pérez, T. G. Folland, I. Errea, J. Taboada-Gutiérrez, J. Duan, J. Martín-Sánchez, A. I. F. Tresguerres-Mata, J. R. Matson, A. Bylinkin, M. He, W. Ma, Q. Bao, J. I. Martín, J. D. Caldwell, A. Y. Nikitin, and P. Alonso-González, Infrared Permittivity of the Biaxial van der Waals Semiconductor α-MoO_3 from Near- and Far-Field Correlative Studies, Adv. Mater. **32**, 1908176 (2020).

[2] J. Martín-Sánchez, J. Duan, J. Taboada-Gutiérrez, G. Álvarez Pérez, K. V. Voronin, I. Prieto, W. Ma, Q. Bao, V. S. Volkov, R. Hillenbrand, A. Y. Nikitin, and P. Alonso-González, Focusing of in-plane hyperbolic polaritons in van der Waals crystals with tailored infrared nanoantennas, Sci. Adv. **7**, eabj0127 (2021).

[3] L. Novotny and B. Hecht, *Principles of Nano-Optics*, 2nd ed. (Cambridge University Press, 2012).

[4] I. V. Rubtsov and A. L. Burin, Ballistic and diffusive vibrational energy transport in molecules, J. Chem. Phys. **150**, 020901 (2019).

[5] N. I. Rubtsova, L. N. Qasim, A. A. Kurnosov, A. L. Burin, and I. V. Rubtsov, Ballistic energy transport in oligomers, Acc. Chem. Res. **48**, 2547 (2015).

[6] P. Hamm and M. T. Zanni, *Concepts and Methods of 2D Infrared Spectroscopy* (Cambridge University Press, Cambridge, UK, 2011).

[7] J. Duan, G. Álvarez-Pérez, C. Lanza, K. Voronin, A. I. F. Tresguerres-Mata, N. Capote-Robayna, J. Álvarez-Cuervo, A. Tarazaga Martín-Luengo, J. Martín-Sánchez, V. S. Volkov, A. Y. Nikitin, and P. Alonso-González, Multiple and spectrally robust photonic magic angles in reconfigurable α-Moo_3 trilayers, Nat. Mater. **22**, 867 (2023).
